# Seroprevalence of bovine brucellosis and associated factors among dairy cows with recent cases of abortion in Ethiopia

**DOI:** 10.1002/puh2.54

**Published:** 2023-07-06

**Authors:** Temesgen Kassa Getahun, Beksisa Urge, Gezahegn Mamo

**Affiliations:** ^1^ Animal Health Research Ethiopian Institute of Agricultural Research Holeta Animal Health Research program Holeta Ethiopia; ^2^ Department of Veterinary Public Health and Microbiology Addis Ababa University College of Veterinary Medicine and Agriculture Bishoftu Ethiopia

**Keywords:** abortion, bovine brucellosis, Ethiopia, risk factors, seropositivity

## Abstract

**Background:**

Brucellosis is a serious zoonotic disease affecting human and all domestic animals. It is considered to be one of the great public health problems all over the world. Little attention has been paid to bovine brucellosis in small holder dairy farm and in animal owners and farm workers in central highland of Ethiopia. This research was conducted with the aim of determining seropositivity and identifying the potential risk factors of brucellosis in dairy cows with recent cases of abortion in the central highlands of Ethiopia.

**Methods:**

This was a cross‐sectional study with purposive sampling carried out on dairy cows in the farms and kebeles from December 2019 to May 2020. The serum samples were initially screened for brucellosis using the Rose Bengal plate test (RBPT) followed by the complement fixation test (CFT) for confirmation. The risk factors for bovine brucellosis were assessed using univariable Firth bias reduced logistic regression.

**Results:**

A total of 352 samples were tested. Serological positivity for bovine brucellosis was detected in 1.2% (95% CI: 0.47–2.97) by RBPT, and bovine brucellosis confirmed by CFT was 0.6% (95% CI: 0.16–2.09). The risk factors for confirmed bovine brucellosis were the late stage of abortion (OR = 1.046, *p* = < 0.0041), retained fetal membranes (OR = 32.74, *p* = 0.006), and market‐based stock replacement (OR = 1.0638, *p* = 0.0008), which are significantly associated at 95% confidence interval.

**Conclusion:**

The proportion of brucellosis among aborted cows seems to be low in the study area. However, given the lack of control strategies in the area, there is a potential risk of transmission in dairy cattle and the human population in the study areas.

## Graphical abstract

Serological positivity for bovine brucellosis was detected in 1.2% by RBPT and bovine brucellosis confirmed by CFT was 0.6% by a cross‐sectional study with purposive sampling from large‐scale, medium‐scale, and smallholder dairy farms. Even though low, a potential risk of transmission in dairy cattle and human.

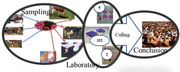

## INTRODUCTION

Brucellosis is a contagious bacterial disease that affects animals globally causing significant economic losses, especially in sub‐Saharan Africa [1]. Although it is almost eliminated from the majority of developed nations, brucellosis is prevalent in Africa, the Middle East, Central and Southeast Asia, Central and South America, and the majority of Southern European countries. However, brucellosis is unreported and underdiagnosed in many developing countries [2].

Bovine brucellosis is caused by *Brucella abortus*, *Brucella Melitensis*, and *Brucella suis*, the Gram‐negative bacteria. They are oxidase, catalase, and urease positive. *Brucella* species are facultative intracellular, Gram‐negative, nonspore‐forming, and partially acid‐fast Coccobacilli that lack capsules, endospores, or native plasmids. They survive freezing and thawing, but most disinfectants are active against *Brucella*. Pasteurization effectively kills *Brucella* in milk [3]. In cattle, the method of transmission involves contact between animals after an abortion and a retained placenta. The organisms are commonly ingested after being exposed to contaminated grassland or livestock barns, although other alternatives include inhalation and conjunctival inoculation. It is also possible to spread an infection when feeding newborn calve pooled colostrum. Sexual transmission has little impact on the epidemiology of bovine brucellosis. However, because artificial insemination (AI) can spread the illness, only animals confirmed to be disease‐free should have their semen collected [4].

Bovine brucellosis is primarily characterized by abortions that occur late in pregnancy. Fetal membrane retention and endometritis are common complications, and the latter may contribute to infertility in subsequent pregnancies. Abortion rates in herds that are entirely vulnerable might range from 30% to 8% [5, 6].

Bovine brucellosis is predominantly a disease of sexually mature animals that are of major economic importance. It affects approximately 5% of the livestock population worldwide, and an increasing proportion of the disease is reported in countries lacking a preventive program. Economic losses result from delayed heat, loss of calves, reduced milk production, culling, and economic losses from international trade bans in the tropics and subtropics [7].

The first reports of the disease in Ethiopia were in the 1970s [8]. In the last two decades, several serological surveys have shown that it is endemic and widespread [9, 10] in cattle in both highland and lowland areas [11, 12, 13]. However, there is limited information on the seroprevalence of bovine brucellosis in medium and smallholder dairy cattle in Holeta town, Wolmera district, which is located in the milk‐shed areas for Addis Ababa and its surroundings. We describe the proportion and risk factors associated with brucellosis in bovines with a recent history of abortion.

## METHODS

### Study design, population, and sampling

A cross‐sectional study was conducted from November 2019 to May 2020. A purposive sampling technique was applied to select bovines with a history of abortion within 1 month prior to survey in medium‐, large‐, and small‐scale farms in Holeta Town, Wolmera district and Ethiopian Institute of Agricultural Research (EIAR) Holeta Agricultural Research Center (HARC) branch Adea Berga dairy farm, Oromia regional state, Ethiopia. The dairy cows under study were pure Holstein Friesian and Jersey breeds, indigenous breeds, and Boran Holstein Friesian and Boran Jersey crossbreeds with no history of vaccination.

The sample size for this study was estimated based on the previous prevalence of 92% in Holeta Town [14], a precision of 5%, and a 95% level of confidence interval.

### Study setting

Holeta is a part of Ethiopia's central highlands and is situated 29 km west of Addis Ababa. The main livelihoods in this region are mixed crop‐livestock farming and dairy production (CSA, 2016). The estimated total number of cattle in the area is 175,741, of which 172,769 (98.3%) are local breeds and 2972 (1.7%) are crossbred cattle. There are 11 medium‐sized dairy farms in Holeta town [15]. The region also hosts the HARC that has 350 pure Jersey, Boran, and Holstein Friesian and Jersey crossbreeds kept under a semi‐intensive rearing system.

### Study variables and data collection

Animal‐related variables considered were species, age, body condition score (BCS), lactation, reproductive status, parity number, period of abortion, and history of abortion. BCS was estimated subjectively using Svendsen [16] guidelines.

To prevent unanticipated harm to humans and to reduce any stress that might be placed on the animals, the dairy cows with a history of recent abortions were adequately segregated and restrained. After cleaning the jugular vein site on each cow, blood samples of 7–10 mL were collected in sterile, plain vacutainer tubes. As instructed in the World Organization for Animal Health manual of diagnostic tests and vaccines [17], the samples were maintained in a slanting position overnight at room temperature to separate the serum. The serum was then carefully transferred into sterile screw‐capped Eppendorf tubes (1.8 mL), labeled, and kept at −20°C in the Animal Health Microbiology laboratory of the EIAR HARC.

The serological tests were performed at the National Veterinary Institute, Bishoftu Ethiopia using the Rose Bengal plate test (RBPT) according to the procedures described by [18] the World Organization for Animal Health [19] and the manufacturer's instructions.

Serum that tested positive for RBPT was then subjected to a complement fixation test (CFT) test using the common Brucella antigen. Sera with a significant reactivity, more than 75% fixation of complement (3+) at a dilution of 1:5, or at least 50% fixation of complement (2+) at a dilution of 1:10 or higher, were classified as positive, whereas complete hemolyses or lacks of fixation were classified as negative.

### Data analysis

Data was coded entered in Microsoft Excel version 10 and exported to R version 4.0. On the basis of RBP and CFT positive, the proportion of seropositivity was calculated by dividing the number of Brucella reactors by the total number of examined animals. The relationship between seropositivity and possible risk factors was analyzed using Chi‐square and Firth bias‐reduced logistic regression (used to reduce the bias when the case number is less than 10% of the sampled population) analysis.

### Ethical considerations

Ethical clearance was obtained from the animal research ethical review committee of the College of Veterinary Medicine and Agriculture, Addis Ababa University Ethiopia (Date: 15/10/2019GC, Ref. No. VM/ER/10/01/12/2020).

## RESULTS

A total of 267 farms were surveyed for this study, 2 large‐, 11 medium‐, and 254 small‐scale farms. A total of 352 recently aborted cows, 222 cross‐ and 130 local breeds, were sampled in the study duration. A total of 137 (38.9%) were bred using AI, 80 (22.7%) were bred using natural mating, whereas 135 (38.3) use both breeding methods. The details of the bovine study sample and the barn characteristics are shown in Table 1.

**TABLE 1:** Characteristics of bovines surveyed for brucellosis in Holeta, Wolmera, and Adea Berga districts, Oromia region, Ethiopia, 2019–2020.

		Rose Bengal plate test		Complement fixation test	
Variables	Total	*n*	(%)	*n*	(%)
**Origin/site**					
**Adea Berga farm**	30	0	0	0	0
**Holeta town**	163	4	(1.2)	2	(0.6)
**Wolmera**	159	0		0	0
**Herd size**					
**Large scale**	58	1	0.3	0	0
**Medium scale**	40	1	0.3	0	0
**Smallholders**	254	2	0.6	2	0.6
**Breed**					
**Cross**	222	3	0.9	1	0.3
**Local**	130	1	0.3	1	0.3
**Young**	78	1	0.3	2	0.6
**Adult**	233	2	0.6	0	0
**Old**	41	1	0.3	0	0
**Parity status**					
**Primiparous**	57	1	0.3	1	0.3
**Pupiparous**	295	3	0.9	1	0.3
**Late stage**	13	3	0.9	2	0.6
**Early stage**	339	1	0.3	0	0
**Source of stock replacement**					
**Government**	1	0	0	0	0
**Own source**	198	3	0.9	1	0.3
**Market purchase**	16	1	0.3	1	0.3
**Both**	52	0	0	0	0
**Hygiene of barn**					
**Poor**	111	2	0.6	2	0.6
**Medium**	140	2	0.6	0	0
**Hygiene of barn**					
**Good**	101	0	0	0	0
**Intensive**	32	0	0	0	0
**Extensive**	164	1	0.3	1	0.3
**Semi‐intensive**	156	3	0.9	1	0.3
**Retained fetal membrane**	48	4	1.2	2	0.6
**Natural mating**	80	0	0	0	0
**AI**	137	4	1.2	2	0.6
**Both**	135	0	0	0	0
**Presence of parturition pen**	7	2	0.6	0	0
**Separation of cow during parturition**	335	4	1.2	2	0.6
**Cleaning of calving area after parturition**	280		0.9	2	0.6

### Seropositivity for brucellosis

There were four bovines 1.2%, (95% CI: 0.47–2.97) that tested positive on RBPT, and two bovines 0.6% (95% CI: 0.16%–2.09%) that tested positive on CFT. Most of sampled cows were extensively managed and replaced from own farms (Table 2).

**TABLE 2:** The farm characteristics of bovines with recent abortion studies for seropositivity for brucellosis in Holeta, Wolmera, and Adea Berga districts, Oromia region, Ethiopia, 2019–2020.

	Large scale farm		Medium scale farm		Small holder farmer	
Farm characteristics	*n*	(%)	*n*	(%)	*n*	(%)
**Type of farming**						
**Extensive**	–	–	–	–	164	(100)
**Intensive**	0		8(25)		24	(75)
**Semi‐intensive**	2 (2.8)		3(4.2)		66	(93)
**Source of stock replacement**						
**Own farm raised**	55 (19.4)		29(10.2)		200(70)	
**Market purchased**	3 (6.1)		10(20.4)		36(73.5)	
**Government gift**	0		1(5.3)		18(94.7)	
**Service type**						
**Artificial insemination**	56 (34.8)		33(22)		48(43.3)	
**Natural mating**	1 (0.9)		2 (1.8)		107(97.3)	
**Both**	0		2 (1.6)		76(97.4)	
**Having parturition pen**	2 (28.6)		2 (28.6)		3(42.9)	
**Cleaning parturition area after birth**	2 (0.75)		2 (0.75)		4(1.49)	
**Hygiene of barn**						
**Good**	30 (29.7)		12 (11.9)		59(59.4)	
**Medium**	28 (20)		23 (16.4)		89(63.6)	
**Poor**	0		5 (4.3)		106(95.5)	
**Repeat breeding**	8 (14.8)		3 (5.6)		43(79.6)	

The seroprevalence of bovine brucellosis in the late stage of abortion (OR = 1.283, *p* = 0.0001), retained fetal membrane (OR = 1.046, *p* = 0.0014), and market source of stock replacement (OR = 1.0638, *p* = 0.0008) were statistically significant, whereas other factors were not statistically significant (*p* > 0.05) (Table 3).

**TABLE 3:** Multivariable Firth's Bias‐Reduced Logistic Regression analysis of risk factors associated with dairy cow brucellosis seropositivity among bovines in Holeta, Wolmera, and Adea Berga districts, Oromia region, Ethiopia, 2019–2020.

		CFT			
Variables	No. of cow tested	*n*	(%)	Adjusted OR (95% CI)	*p* Value
**Stage of abortion**					
**Early stage**	339	0	0	1	
**Late Sage**	13	2	0.6	1.283 (1.215–1.3557)	0.1
**RFM**	48	2	0.6	1.046 (1.0187–1.0754)	0.0014
**Source of stock replacement**					
**Government**	1	0	0	1	
**Own source**	198	1	0.3	6.549 (30.066–76.357)	0.075
**Market Purchase**	16	1	0.3	1.0638 (1.026–1.1029)	0.0008
**Both**	52	0	0		0.399
**Presence of parturition pen**	7	0	0	1	0.281

Abbreviations: CFT, complement fixation test; CI, confidence interval; OR, odds ratio.

Thus, the reduced model revealed that cows with late‐stage abortion, retained fetal membrane, and market purchase herd replacement were 1.283, 1.046, and 1.0638 times more likely to be seropositive to Brucella infection, respectively, than those with early‐stage abortion, without retained fetal membrane, and own or government source of herd replacement (Table 3).

## DISCUSSION

The present study revealed that the overall seroprevalence of *Brucellosis* in the study areas was 0.6%. The seroprevalence in this study was slightly higher than the finding of Yayeh et al. [20], who reported an overall prevalence of 0.14% in the North Gondar Zone, Bashitu et al., [21] 0.2% in Ambo and Debreberhan, and Edao et al., [13] 0.06% in the Addis Ababa area. However, in other ways, Alem and Solomon [22] and Belihu [23] failed to find any seroreactive cattle in the Eastern and Western Showa zones of central Ethiopia and in intensive dairy farms in the Addis Ababa area, respectively. This variation in seroprevalence might be seen due to the difference in the study animal management system. Most of the reactive animals in our study were from smallholder farmers kept under an extensive management system. The dependency of most of the farmers on outside sources for stock replacement could be one possible way of introducing the disease into unaffected herds. This could also be due to differences between the study areas regarding conditions that could facilitate the rate of transmission of the disease [24].

The finding of our study was in close agreement with the findings of Tesfaye [25] (0.69%), Tolosa [26] (0.77%), Gumi et al. [27] (0.9%), and Adugna et al. [28] (1.0%) from Ethiopia and Kang'Ethe et al. [29] (1.0%) from Kenya. On the other hand, there were reports with a relatively higher seroprevalence of bovine brucellosis in other parts of the country: Berhe et al. [9] (3.19%); Kebede et al. [30] (11.0%); Jergefa et al. [31] (2.9%); Haileselassie et al. [32] (4.9%); Ibrahim et al. [10] (3.1%); Degefu et al. [33] (1.38%); Megersa et al. [34] (3.5%); Asmare et al. [35] (1.9%); Gebawo et al. [36] (4.3%); Geresu et al. [8] (1.4%). Similarly, relatively higher seroprevalence were reported in other African countries by other authors: Overall, 8.5% by Omer et al. [37] from Eritrea, 24.5% by Angara et al. [38] from Sudan, 24.0% by Matope et al. [39] from Zimbabwe, and 5.5% by Mai et al. [40] from Nigeria were some of the reports.

The observed variation in prevalence might be explained by variations in production systems and animal care. The majority of previous research studies indicating higher prevalence, which varied authors reported, were carried out in intensively managed herds where cattle from numerous owners gathered at grazing or watering places. The findings of this study showed that all of the confirmed instances came from smallholder dairy farmers who kept their dairy animals under extensive management. Therefore, the reasons for the low prevalence of bovine brucellosis in these study areas could potentially be explained by improved hygiene practices, the separation of cows during parturition, cleaning and disinfection activities, the culling of infected animals, relying on their own herd to replace stock in 2 large‐scale farms and 11 medium‐scale farms, and the prevailing management differences between intensive, semi‐intensive, and extensive production systems. In order to avoid interaction with animals, the farms’ general good hygiene standards and practices are also indicative of this.

In addition to the estimation of seroprevalence, this study was also carried out to assess the risk factors associated with disease occurrence. The previous history of abortion stage has a statistically significant association with the seropositivity of bovine brucellosis. This was in agreement with previous reports by Minda et al. [41]. This could be explained by the presence of higher seropositivity in cows in the last trimester, which may be due to the preferential localization of Brucella in the uterus, in which allantoic fluid factor and erythritol stimulate the growth of Brucella in the uterus and increase in the placenta and fetal fluid from about the fifth month of gestation [42, 43].

In the current study, there was also a highly significant association between seropositivity for brucellosis and cows having a history of retained fetal membranes. Following a brucellosis‐related abortion, the placenta is frequently retained, and the uterine wall becomes inflamed (metritis). Aparicio [44] reported that *Brucella*‐infected cows were expected to abort three‐to‐four times more than unexposed cows. This could also be explained by the fact that retained fetal membrane is a typical outcome of brucellosis. Other studies have also shown a significant association between seropositivity and retained fetal membrane [9, 10, 26, 45–47].

There were statistically significant differences in the seroprevalence of brucellosis seropositivity and breeding methods. In the present study area, most farms used AI (38.9%) more often than bulls (22.7%) for breeding purposes. There was a higher seroprevalence rate in the AI service, whereas there was no seropositive record in the bull mating method. The sources of replacement stock were shown to significantly affect the prevalence of bovine brucellosis in study areas (*p* = 0.0008). Those animals purchased from other areas were relatively more susceptible to brucellosis than cattle grown and replaced the stock.

According to the current study, *Brucella* infection did not show significant variation between breeds. The present finding agrees with the previous reports of Kebede et al. [30]; Amenu et al. [48]; Asmare et al. [35]; Minda et al. [41], who reported that the seropositivity of *Brucella* infection was independent of the breeds. The variation in the number of animals sampled per breed group might be responsible for the absence of significant variation in *Brucella* infection between the breeds. Crossbreds, on the other hand, were becoming more common in our study area, and farmers treated crossbred cows better than local breeds.

According to the present study, there was no statistically significant difference among age groups for *Brucella* seropositivity. All positive cows (0.6%) were found in the adult age group, whereas no *Brucella* seropositivity was observed in the young and old age groups of dairy cattle in the study sites. Similar findings were also reported by Jergefa et al. [31], Amenu et al. [48], Geresu et al. [49], where age was not significantly associated with *Brucella* seropositivity.

The higher seroprevalence of brucellosis among adult cows may be related to the higher number of adult cows included in the study. In addition, it may be related to their advanced age as the organism may remain latent or chronic for an unspecified period before manifesting as a clinical disease. The other justification is also possible as age is one of the intrinsic factors that influence the susceptibility to *Brucella* infection. Brucellosis appears to be more associated with sexual maturity [24]. It is essentially a disease of sexually mature animals and susceptibility increases with sexual maturity and pregnancy due to the influence of sex hormones and placenta erythritol on the pathogenesis of brucellosis [43]. On the other hand, younger animals tend to be more resistant to infection and frequently clear infections, although latent infections can occur [50].

Based on the current study findings, the following recommendations are forwarded to curb further spread of the disease in both cattle and human populations:

- Replacement stock should be purchased from herd known to be free of brucellosis.
- Strict movement control of animal from one area to another in order to prevent the spread and transmission of the disease from infected cattle to the noninfected ones.
- The implementation of test and slaughter policy with compensation payment to the farmers as the prevalence of the disease is low in the study area.

## CONCLUSION

In Holeta town, Wolmera district, and HARC Adea Berga dairy farm in West Shoa, Oromia Region Ethiopia, the overall seroprevalence of bovine brucellosis with recent abortion history was 0.6%. The finding of positive serological reactors did not only suggest the presence of the disease in the cattle population in the areas but also indicated the presence of foci of infection that could serve as sources of infection for the spread of the disease into unaffected animals and humans. In this finding, the stage of abortion, retained fetal membrane, the source of stock replacement, and breeding methods were statistically significant risk factors associated with dairy animal brucellosis seropositivity.

## AUTHOR CONTRIBUTIONS

*Conceptualization; data curation; formal analysis; investigation; methodology; writing—original draft; writing—review and editing*: Temesgen Kassa Getahun. *Resources; supervision; writing—review and editing*: Beksisa Urge. *Resources; supervision; validation; visualization; writing—review and editing*: Gezahegn Mamo.

## CONFLICT OF INTERESTS STATEMENT

No conflict of interest between authors.

## ETHICS STATEMENT

An ethical clearance certificate was obtained from the animal research ethical review committee of the College of Veterinary Medicine and Agriculture (Date: 15/10/2019GC, Ref. No. VM/ER/10/01/12/2020) based on the assessment of the research proposal. The standard ethical principles and conduct were implemented for animal study participants. Written and oral informed consents were obtained from livestock owners.

## Data Availability

Data for this publication can be available when required
